# Supplementary material for: Genome-Wide Identification, Phylogenetic Evolution, and Abiotic Stress Response Analyses of the Late Embryogenesis Abundant Gene Family in the Alpine Cold-Tolerant Medicinal Notopterygium Species
Source: Int J Mol Sci. 2025 Jan 9;26(2):519. doi: 10.3390/ijms26020519 (PMC11765234; doi:10.3390/ijms26020519)
Supplement: Supplementary file 1 [file ijms-26-00519-s001.zip › Supplementary Fig. S1-S7.pdf]

Genome-wide identification, phylogenetic evolution and abiotic stress response analyses of *LEA* gene family in the alpine cold-tolerant medicinal *Notopterygium* species

Figure captions:

Fig. S1 Evolutionary tree of LEA genes in *Notopterygium* species

Fig. S2 Statistical chart of *cis*-acting elements quantity

Fig. S3 Venn diagram of DEGs in various tissues of *N. franchetii*

Fig. S4 Venn diagram of DEGs in various tissues of *N. incisum*

Fig. S5 UpSet plot of DEGs in various tissues of *N. forbesii*

Fig. S6 UpSet plot of DEGs in various tissues of *N. oviforme*

Fig. S7 UpSet plot of DEGs in roots of four *No*topterygium species

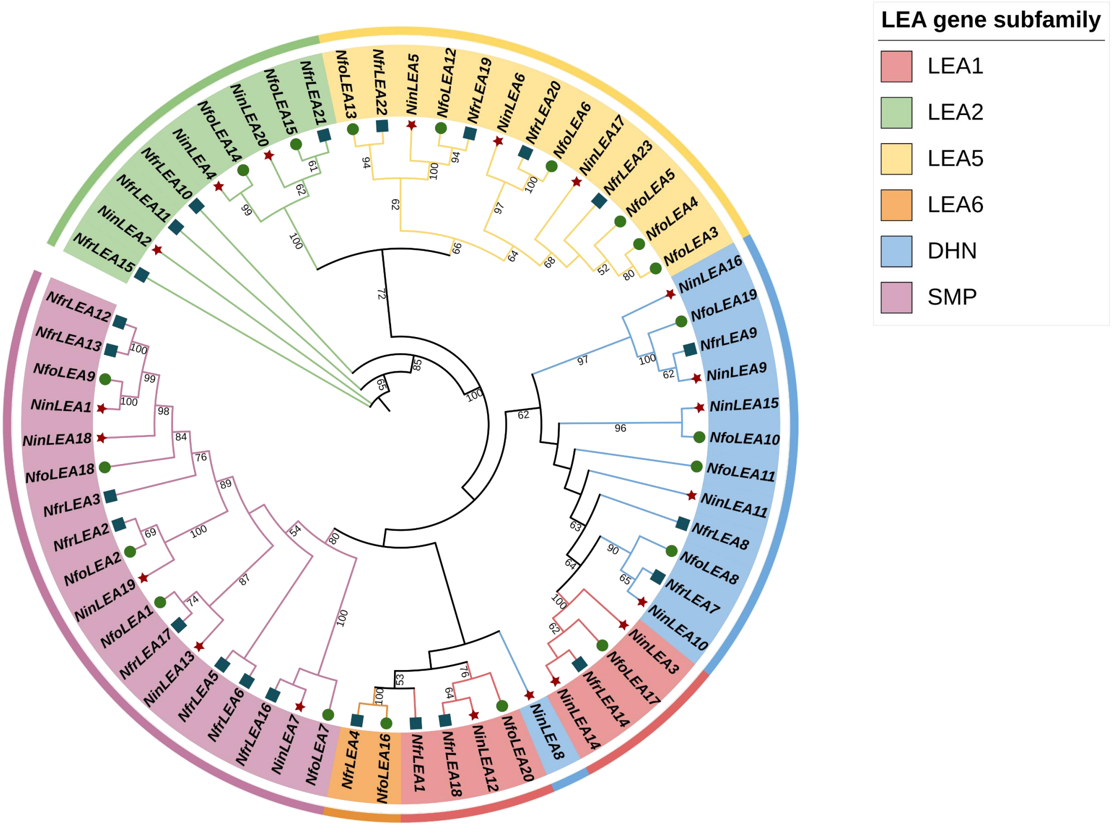

Fig. S1 Evolutionary tree of LEA genes in *Notopterygium* species

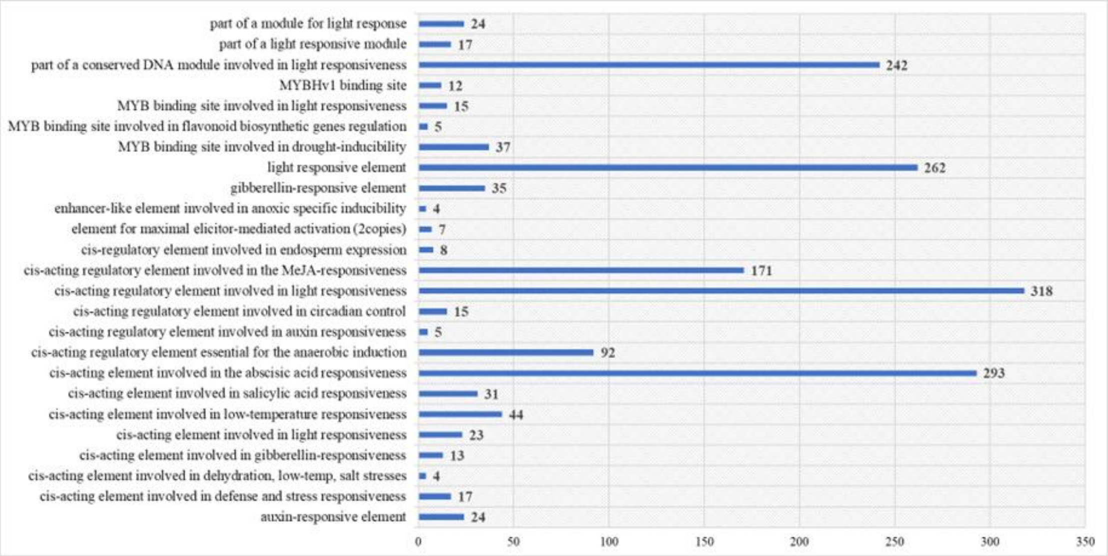

Fig. S2 Statistical chart of cis-acting elements quantity

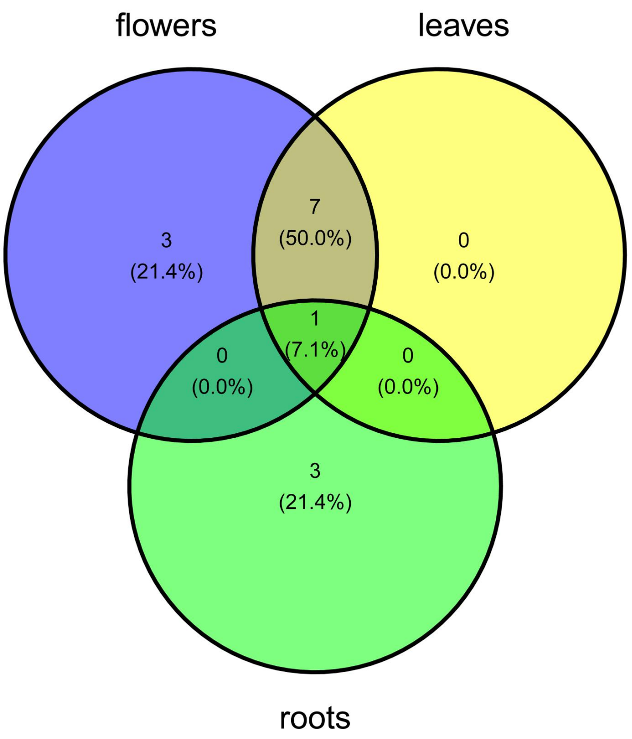

Fig. S3 Venn diagram of DEGs in various tissues of *N. franchetii*

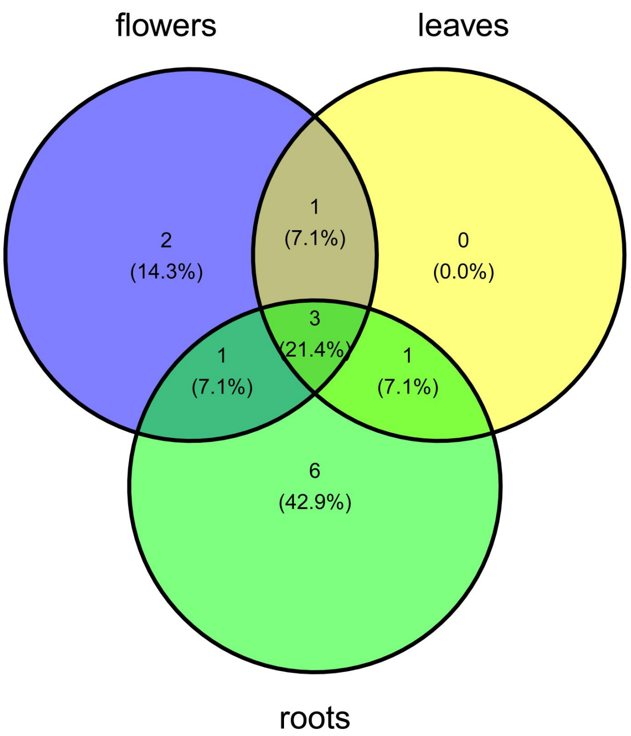

Fig. S4 Venn diagram of DEGs in various tissues of *N. incisum*

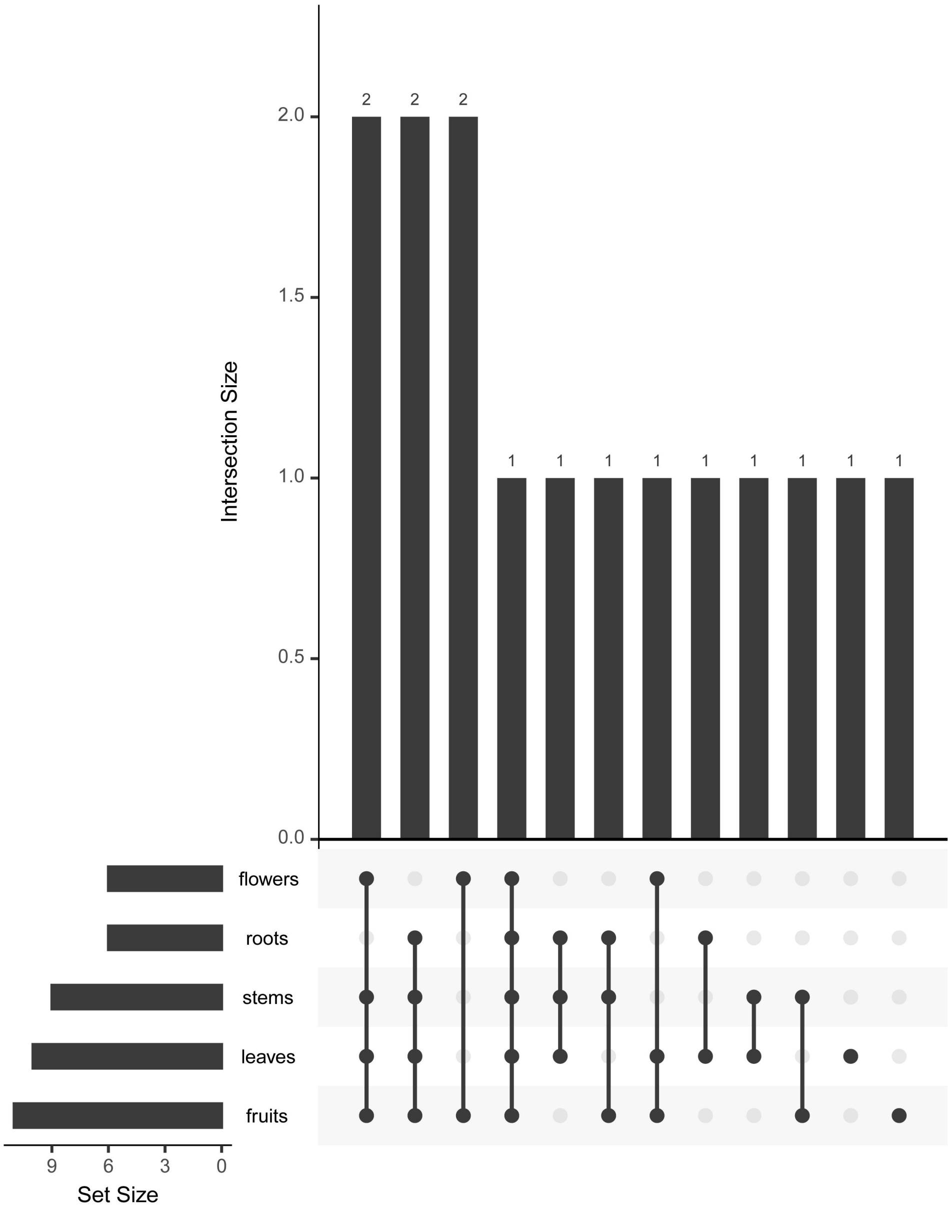

Fig. S5 UpSet plot of DEGs in various tissues of *N. forresii*

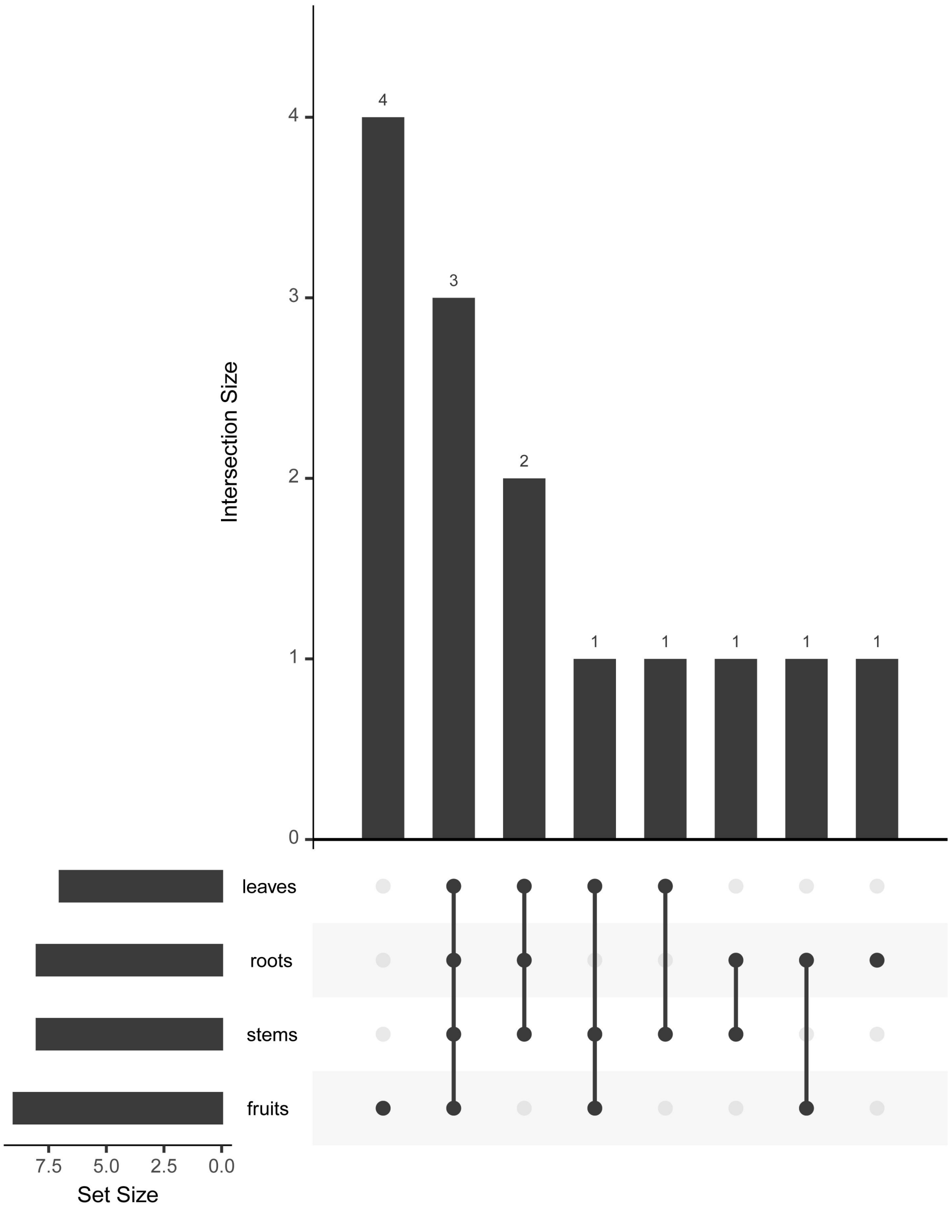

Fig. S6 UpSet plot of DEGs in various tissues of *N. oviforme*

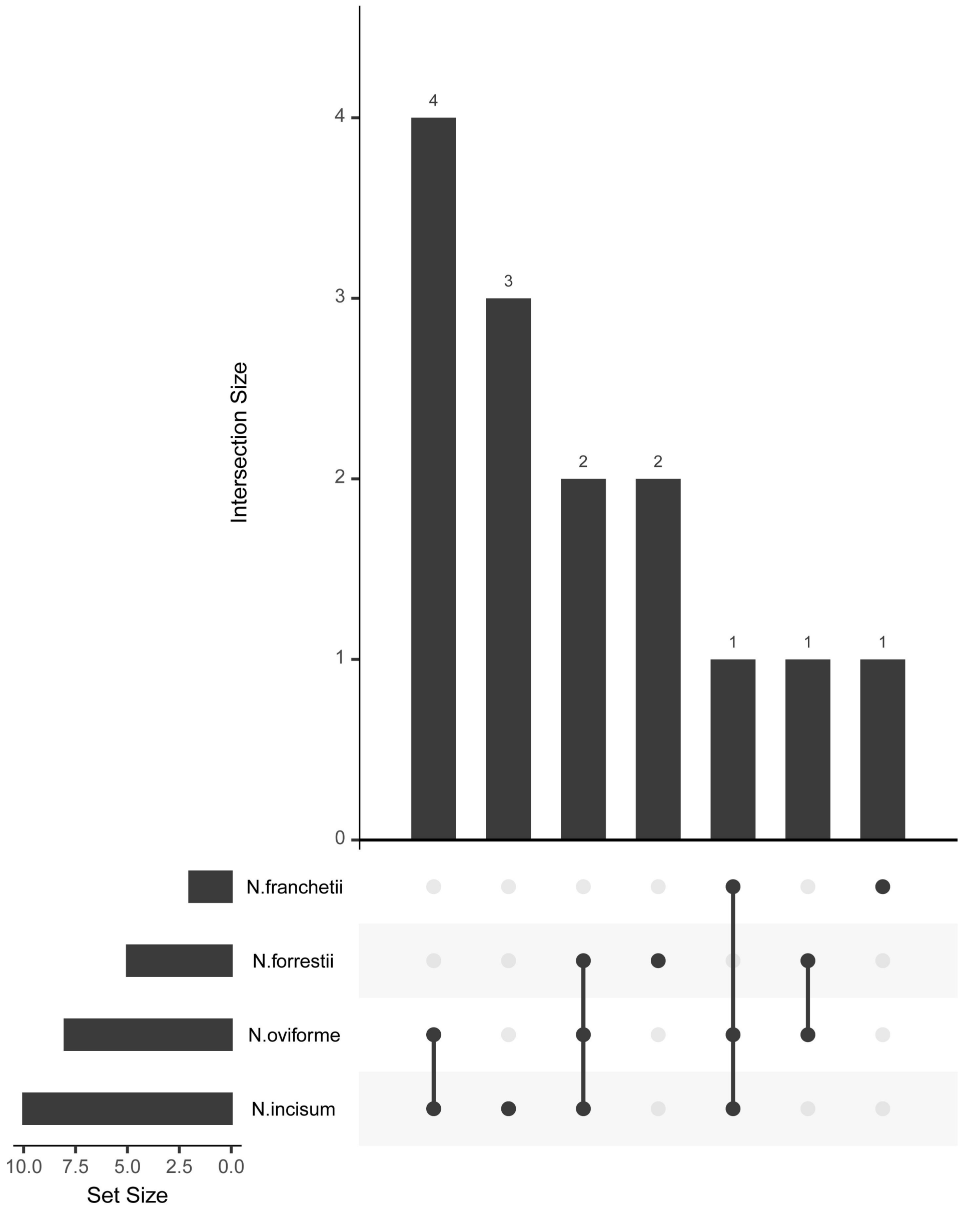

Fig. S7 UpSet plot of DEGs in roots of four *Notopterygium* species
